# Cultural and religious influences on shared decision-making in treatment escalation planning with older adults in the acute setting: a qualitative secondary analysis of interview studies seeking clinician and patient perspectives

**DOI:** 10.1093/ageing/afaf357

**Published:** 2025-12-17

**Authors:** Alya A Khashaba, Stephen J Brett, Bronwen E Warner

**Affiliations:** Department of Surgery and Cancer, Imperial College London, London, UK; Department of Surgery and Cancer, Imperial College London, London, UK; Directorate of Critical Care, Imperial College Healthcare NHS Trust, London, UK; Department of Surgery and Cancer, Imperial College London, London, UK

**Keywords:** culturally competent care, person-centred care, shared decision-making, treatment escalation planning, qualitative research, older people

## Abstract

**Background:**

Treatment escalation plans guide medical interventions during acute health deterioration. Professional guidance encourages shared decision-making in treatment escalation planning in the United Kingdom (UK). Cultural and religious beliefs are likely to influence patients’ preferences for severe illness.

**Aim:**

To explore patients’ and clinicians’ perceptions of cultural and religious influences on shared decision-making in treatment escalation planning for older adults.

**Methods:**

This study was a qualitative secondary analysis of two UK-based primary datasets exploring clinician and patient perspectives of shared decision-making in treatment escalation planning with older adults. Interviews were included where participants volunteered cultural and religious influences on treatment escalation decision-making (20 clinicians, 11 patients). Reflexive thematic analysis was conducted.

**Results:**

Themes from clinician interviews were: ‘Decisions informed by a “British medical way”’, ‘Cultural and clinical dissonance creates tension in treatment escalation planning discussions’ and ‘Convincing and compromising: negotiating a culturally acceptable decision’.

Themes from patient interviews were: ‘A relational decision made within a family network’, ‘Expectations built on experiences in different cultural contexts’ and ‘Expert plan, Divine outcome’.

**Conclusions:**

Clinicians and patients approached the role of culture in treatment escalation discussions from distinct positions. Clinicians drew on shared professional norms and considered different cultural attitudes potentially challenging to notions of clinical benefit and individual autonomy. Patients described an approach centred on family, prior healthcare experiences and faith. Recognising these differing priorities helps identify potential challenges during treatment escalation planning and areas for enhancing current shared decision-making guidance to better support culturally sensitive care.

## Key Points

- Shared decision-making is encouraged in UK guidelines for treatment escalation planning; cultural and religious beliefs shape care preferences in severe illness.
- This qualitative secondary analysis integrated clinician and patient perspectives from two UK-based primary studies.
- A clinically focused ‘British medical way’ is juxtaposed with cultural emphasis on family, faith and lived healthcare experience.
- The research offers insight into patient priorities; relational autonomy may better support person-centred care in some instances.
- This analysis contributes to deliberation around guidelines balancing clinical goals, cultural values and equitable resource use.

## Introduction

In a multicultural society like the United Kingdom (UK), clinicians routinely encounter patients from diverse cultural and religious backgrounds [1–3]. Conceivably, cultural and religious beliefs contribute to how older patients and their families understand and navigate severe illness, the approach of death, and dying [4–9] and may influence their interactions with healthcare professionals and expectations of intervention [9]. Professional guidance therefore recognises the importance of integrating cultural and religious needs in providing person-centred care, particularly when transitioning to end-of-life care [10–12].

Older adults represent a high proportion of acute hospital admissions [13, 14]. Complex comorbidities increase risk of deterioration with uncertain outcomes [15, 16]. Advances in life-prolonging medical therapies raise questions about how to select treatments aligned with individual values [17]. Treatment escalation plans (TEPs) detail interventions, such as organ support and cardiopulmonary resuscitation, to be provided if a patient acutely deteriorates in hospital [18].

Shared decision-making (SDM), incorporating clinician expertise and patient preference, is encouraged in TEP discussions in the UK [11, 19–21]. However, it is not clear whether SDM is truly feasible in TEPs, due to lack of shared understanding about what treatments can achieve, uncertainty inherent to the decision, finite healthcare resources and the sometimes-insoluble nature of subjective outcomes [22, 23].

In this study, we explored how patients and clinicians perceive cultural and religious influences on shared decision-making for treatment escalation planning for older adults.

## Methodology

### Study design

This study is a qualitative secondary analysis of two datasets [22, 23]. Primary analyses explored clinician and patient perspectives of SDM in TEP with older adults in the UK acute setting. Although cultural and religious influences were not a focus of the primary research question, participants frequently introduced these ideas in response to open questions. This observation implied their significance within personal narratives around TEP decisions and prompted the in-depth secondary analysis presented here. Interview topic guides used in the primary studies are included in Appendices S1 and S2.

The primary datasets comprised transcripts of semi-structured interviews with 26 clinicians and 32 patients. Clinicians were consultants or registrars in emergency medicine (EM), general internal medicine (GIM), intensive care medicine (ICM) and palliative care (PC)*.* Patients were aged ≥65 years, with purposive sampling for age, frailty and ethnicity [24, 25]. Full details of the primary studies are reported separately [22, 23].

### Data analysis

Shared decision-making was considered a social construct, viewed through a constructivist epistemology in which participants and researchers collaboratively generate meaning from their lived experiences to understand the nature of decision-making and deliberate on what ‘good’ decision-making is. Reflexive thematic analysis, as per Braun and Clarke [26], was used to explore patterns of meaning as themes. Analysis was inductive, to remain open to new data-driven insights.

The full primary datasets (26 clinician interviews and 27 patient interviews involving 32 patients) were initially explored to identify ideas about cultural and religious influences in decision-making. Interviews where self-reported cultural and religious influences (from participants of any background or belief) were expressed in sufficient depth were thereafter included in the secondary analysis (20 clinician interviews and 9 patient interviews involving 11 patients). The analytical phases are described in Appendix S3.

To ensure transparency and provide context for our interpretation [26, 27], all researchers engaged in consistent reflexive practice, and the analytical process was documented and reported in depth.

Analysis is reported according to The Big Q Qualitative Reporting Guidelines [28].

### Reflexivity

All authors are clinical academics. The primary researcher and interviewer, B.E.W., is a physician undertaking a PhD. The project supervisor, S.J.B., is an ICM consultant with an academic interest in medical decision-making*.* BEW and SJB have prior experience in the conduct and analysis of qualitative research in a clinical setting.

A.K. joined the team as a secondary analyst after data collection was completed, with no prior relationship to the primary team or study participants. Regular meetings between A.K. and B.E.W. provided context and insights into the data collection process and participant interactions. A.K. and B.E.W. reflected on how B.E.W.’s background as a White British clinician and an interviewer may have influenced interview dialogue about culture and religion. A.K. was aware that her positioning and beliefs as an immigrant Egyptian Muslim woman and a UK medical graduate may affect her interpretations.

Secondary analyst reflexivity is described further in Appendix S4. Primary researcher reflexivity is published separately [22, 23, 27].

### Ethics

Ethical approval for the primary clinician and patient studies was granted by the Health Research Authority (22/HRA/4387) and South Central—Hampshire A Research Ethics Committee (22/SC/0476), respectively. Participants gave written and verbal consent to participate in a study designed to understand important considerations in SDM in TEP. Participants frequently volunteered ideas about cultural and religious influences on TEP, despite these not being the focus of the primary research question. This analysis was therefore deemed to be within the scope of the primary consent, and no further consent was sought. Secondary research question development and subsequent analysis were performed with respectful intent [29, 30]. Pseudonymised interview transcripts of the full datasets were made available by the primary research team (B.E.W. and S.J.B.) to A.K. for secondary analysis, and participant anonymity and confidentiality were maintained throughout.

## Analysis

### Clinicians’ perspectives

Clinicians’ characteristics are presented in Table 1.

**Table 1:** Clinicians’ characteristics

Grade specialty	Consultant	Registrar	Total
EM	3	1	4
GIM	5	2	7
ICM	3	3	6
PC	3	0	3
Total	14	6	20

There were three themes: ‘decisions informed by a “British medical way”’, ‘cultural and clinical dissonance creates tension in TEP discussions’ and ‘convincing and compromising: negotiating a culturally acceptable decision’. Table 2 includes themes and theme definitions. Table 5 includes selected illustrative quotations; an extended version is in Appendix S5.

**Table 2:** Themes and theme definitions from clinician interviews

Theme	Theme definition
1. Decisions informed by a ‘British medical way’	Clinicians have constructed a culturally informed way of formulating a TEP from a medical perspective, according to previous experience, guidance from regulatory bodies and societal norms.
2. Cultural and clinical dissonance creates tension in TEP discussions	Clinicians described tension and moral distress when attempting to reconcile clinically informed escalation decisions with culturally informed patient demands for further escalation.
3. Convincing and compromising: negotiating a culturally acceptable decision	Clinicians described employing significant efforts to communicate with and convince of the medical decision those patients demanding treatment escalation based on cultural or religious priorities. However, clinicians described that they may ultimately concede the final decision to preserve the doctor–patient relationship.

### Themes

#### Theme 1: Decisions informed by a ‘British medical way’

Clinicians from diverse cultural backgrounds often appeared motivated by what they described as ‘a right decision’, based on a British medical culture informing a collective sense of accepted normative ethical practice. This culture was difficult to define but appeared to be subliminally influenced by medical education, law, policy and guidance from regulatory bodies, along with prior experience of what might be acceptable in a particular context. According to this approach, reversible pathology was treated, but when no further benefit was anticipated, there was a shift to symptom-focused care. Individual autonomy was prioritised over familial involvement in patients with capacity. Clinicians described moral distress when challenged by patients with different priorities, feeling that overtreatment or futile intervention conflicted with their ethical obligation for non-maleficence and concerned that deviating from accepted practice would be viewed critically by colleagues.

‘So I think why we find this difficult, and why we sometimes find cultural differences difficult, is because we, as doctors in Britain, have also had a very specific cultural upbringing. So what we think is right is what we learnt at medical school, which is, you treat your reversible things, and then when it becomes futile you take out the cannula and you give them morphine. But that is so specific to being in Britain, at this time, being educated in this way. And actually, even compared to our colleagues in Belgium and Holland, who do euthanasia and assisted dying, we’re quite offended by that, by the idea of euthanasia. And we’re also offended by never palliating, so we’re on a very specific spectrum. The same way that the Belgians are quite… More confident about euthanasia and, say, countries that don’t do palliative care are appalled by it. So I think part of the challenge is us feeling that our way, and the way we practice here, and the way we’ve been brought up, and of course our GMC guidance, and our end-of-life guidance, is the only way. And sometimes it can become a challenge to argue that to the patient who’s saying, but it’s not our way, this is not what we want. That’s an ethical challenge. I don’t really know the answer.’ —GIM Consultant

Nonetheless, many clinicians considered understanding patients’ religious and cultural beliefs to be an important part of providing good care according to contemporary standards. It appeared difficult to reconcile duties to patient-centred care with TEP decisions that clinicians deemed best for the patient.

Some clinicians described feeling more comfortable deviating from local norms and following patients’ cultural preferences, but were aware that this approach differed from local norms. Their reflections contributed analytic insight into the concept of a ‘British medical way’. One clinician who previously worked in Arab countries where TEP decisions were patient-led reported that they more readily prioritised the patients’ decisions over their own; another explained that their religious beliefs made them more ‘soft’ than colleagues:

‘And as a consultant, I certainly think I'm more soft, I'm certainly softer than a lot of my colleagues and I would admit patients that my colleagues wouldn’t admit. And I think the fact religious beliefs, for me, is a big factor, and so I often feel like we don’t have the decision to make God’s decision, and so I'm very religious and that is a big factor for me.’ —ICM Consultant

#### Theme 2: Cultural and clinical dissonance creates tension in TEP discussions

Cultural differences with patients and families about TEP were perceived to be a difficult and emotive issue for all parties. Clinicians sometimes volunteered cultural differences in response to probing questions designed to understand how clinicians navigate conflict around patient requests for more intensive treatment than the clinician believed to be appropriate. Similarly, clinicians’ argument for symptom-focused care could be interpreted by families as neglect. Clinicians sometimes anticipated that patients from certain groups would have challenging views.

‘I think it comes down to a spiritual kind of thing. People want to preserve life for as much as possible. I think a lot of people come from a very strong religious background. I think in most religions, especially you see with Muslim patients, and Orthodox Christians. And maybe the really religious Hindus and Jewish people that they generally want to preserve life as much as possible. And if you give them that option that this one can bring you back to life, even if it’s a slim chance, then that’s the option they’ll take. I think people don’t want to be the ones deciding their life’s fate. They want everything possible and then God to make that decision.’ —GIM Registrar

Another source of tension was the role of families in decision-making. Clinicians noted that patients from some cultural and religious backgrounds commonly relied on familial input, and disagreements often occurred with relatives rather than the patients themselves. Clinicians were torn between a duty to individual patient autonomy and respecting patients’ situatedness in their community.

‘we have to follow the GMC, which is the patient is the most important person to you..., I find it very jarring.’ —GIM Consultant

Resource constraints within a publicly funded NHS added further complexity when patients requested, on cultural grounds, treatment that clinicians deemed excessive. This appeared to be a contentious and unresolved issue for many clinicians.

‘The problem is if there is a chance that you’d survive, even if it’s a much reduced quality of life, and that’s what the patient would want, I find it difficult to not offer that. Because why wouldn’t we offer that? What is it that we’re losing out on by not offering that? If they feel that they would want it, it’s resources. But I’m not sure we’re the people who should be limiting resources. Because if resources should be limited and inform those decisions, there ought to be clear guidance how we do that. So if you think there is some chance of recovery, and the family say, we would be… Or the patient, more importantly, says, yes, I would be happy to be alive at any cost. Then I think it’s difficult not to offer it.’ —ICM Consultant

#### Theme 3: Convincing and compromising: negotiating a culturally acceptable decision

Clinicians appeared to feel deeply uncomfortable about modifying the medical TEP. They described various strategies to achieve acquiescence following conflict based on cultural disagreements: challenging strongly held religious beliefs to understand the reasoning behind patients’ demands for aggressive treatment, framing the medical decision as a non-negotiable decision informed by the patient’s best interests, communicating the irreversibility of the clinical condition, providing second opinions from senior decision makers or involving religious leaders for spiritual support. The motivation behind employing these strategies seemed to be to convince the patient of the medical decision, rather than to understand and incorporate their opinion in the decision-making process.

‘I really don’t think there’s a clear answer to that. I think it’s about having that discussion as much as possible, revisiting it again at a later date, giving people some time to think over it, and discuss it with their family, offering them an opportunity for a second opinion. I don’t know whether we have this. If it’s religious views that are holding them back, then offering them the chance to speak to chaplains and so on about this. But it’s a tricky scenario. And I think the outcome may still be very similar in the sense that you do give it a halfway house approach. And if they end up in that scenario, you’d try CPR for a bit and then call it off after a couple of cycles.’ —GIM Registrar

Some clinicians were committed to enforcing the medical decision. Others were reluctant to cement a plan directly opposing a patient or family’s views and described compromising on the medical decision to accommodate cultural and religious preferences. They described a wish to avoid conflict and commitment to maintaining a trusting doctor–patient relationship. Some believed that prioritising the family’s preferences (especially in cases of prognostic uncertainty, and provided the treatment didn’t cause harm) aligned with a duty to support patients and families through serious illness.

## Patients’ perspectives

Table 3 describes patient characteristics. All but one patient reported having been born in another country and later moving to the UK. Some patients reported specific religious beliefs (such as Judaism, Christianity or Islam), while others indicated belief without specifying a religion by mentions of ‘God’ or ‘prayer’.

**Table 3:** Patients’ characteristics

			Number of participants
Age (years)	65–74		3
	75–84		6
	85+		2
Frailty	None		3
	Mild		4
	Moderate		4
	Severe		0
Sex	Male		5
	Female		6
Ethnicity^a^	Asian or Asian British	*Indian*	4
		*Any other Asian background*	1
	Black, Black British, Caribbean or African	*African*	2
	White	*Any other White background*	1
	Other ethnic group	*Arab*	1
		*Any other ethnic group*	2
Religious belief	Jewish		1
	Christian		2
	Muslim		4
	Indicated but unspecified		2
	Unreported		2

^a^According to ethnic groups defined in the 2021 Census of England and Wales. Ethnic groups not captured are not included in the table, for readability.

There were three themes: ‘a relational decision made within a family network’, ‘expectations built on experiences in different contexts’ and ‘expert plan, Divine outcome’. Table 4 includes themes and theme definitions. Table 5 includes selected illustrative quotations; an extended version is in Appendix S5.

**Table 4:** Themes and theme definitions from patient interviews

Theme	Theme definition
1. A relational decision made within a family network	Patients perceived involving family members as culturally fundamental in decision-making for treatment escalation planning, and highlighted familial contributions in communication and care.
2. Expectations built on experiences in different cultural contexts	Some patients who have moved to the UK from elsewhere had expectations of the roles of healthcare providers and perceptions of Western healthcare, based on experiences in other settings.
3. Expert plan, Divine outcome	Patients trusted that clinicians have the expertise to make the best possible medical decision, but patients with religious beliefs also believed that the ultimate outcome would be dependent on God’s will.

### Themes

#### Theme 1: A relational decision made within a family network

##### 1.1: Making a personal decision is an interpersonal process

Patients highlighted the cultural significance of familial involvement in decision-making and facilitating communication between the patient and the clinical team.

Some patients, particularly from African and South Asian backgrounds, highlighted the cultural significance of actively involving family members in making and communicating treatment decisions. Some specified a cultural tradition of nominating a family member to act as a trusted advocate for the patient and be a comforting presence when difficult news was being communicated.

‘It’s a hard conversation, and it’s a conversation that we do better through a family member, through a very strong family member.’ —Female, aged 65–74, no frailty, African ethnicity

Patients regarded TEP decisions as a heavy burden for clinicians to bear alone and felt that patients and families should share the responsibility in case of subsequent blame.

‘Personally, if there are more than one adult in the family, they should sit down and they should make a decision. Because they don't want to blame the doctors for the rest of their life that he killed my mother or he killed my father. So this is my opinion. Doctors, as I said earlier to you, they always explain what is happening. And they always say pros and cons. This can happen. That's how they speak. This can happen. I'm not saying it will happen. So I think the final decision has to be... the family.’ —Female, aged 75–84, mild frailty, Indian ethnicity

##### 1.2: Familial caregiving as a cultural ideal

Patients highlighted the cultural value of family members providing care.

Some patients felt that physical and remote familial support were important in healing from illness.

‘At least I have got five or six telephones calls every week from my family, encourage me to live on. Come on, we are going to see you, we are missing you, this and that. That motivates me to live, and use my medicine properly, and get well and go home for a few days or a few weeks. That’s very important. But if you are alone in this country, you have got your wife and two kids, that’s it, you don’t have the whole family around you… And we are very family-orientated compared with here.’ —Male, aged 75–84, moderate frailty, any other ethnic group

Several patients described a cultural imperative that family would provide care after hospital discharge. Some were concerned about becoming a burden to their family members, but there was also reluctance to receive outsourced care, due to concerns about trust, perceived disinterest and invasions of privacy. These factors could influence ideas about TEP decision-making and acceptable health outcomes.

**Participant** *: ‘Saying that, I know that you do get sometimes nurses who go and put the medications and everything. But, I don't think that when the nurses go, they are that welcome in any families. It's always... First few days are fine. But when the nurse or whoever is coming for helping every day, it seems that, oh, maybe she knows too much about the family..’.*  **Interviewer***: ‘It’s an invasion of privacy?’*  **Participant***: ‘Exactly. And that happens in our Indian family a lot. And this is not a prejudice statement …. This is just a statement. Not just our Indian people, any Eastern people from Pakistan. Because that's our culture.’ —Female, aged 75–84, mild frailty, Indian ethnicity*

#### Theme 2: Expectations built on experiences in different cultural contexts

Some participants who migrated to the UK as adults reported differences between healthcare in resource-limited settings and in the UK. They perceived Western clinicians to be experts working in well-resourced environments who persist with treatment until patients refuse further intervention. In contrast, participants noted that in the Global South, patients often die at relatively younger ages despite receiving all available treatments, a consequence of the limited resources available. Therefore, patients perceived that providing the best possible care was synonymous with attempting every life-sustaining or life-prolonging treatment. Ideas of ceilings of care therefore seemed unnatural coming from doctors and were instead seen more intuitively to come from the patient’s family after recognising that the treatment being provided led to no further benefit.

‘It's very difficult to decide. And I'm so happy that in this country, doctors don't give up until the patient gives up. They try all the things that are there to save the person. and you can feel very proud of them. Honestly, they really go out of their way to make him better and do things for them. But sometimes you feel that, no, let the patient go because the patient will be a cabbage.’ —Female, aged 75–84, mild frailty, Indian ethnicity

#### Theme 3. Expert plan, divine outcome

Participants from different backgrounds expressed trust in doctors as knowledgeable medical authorities, capable of making appropriate decisions based on the clinical picture. If trusted clinicians explained that minimal consciousness or significant disability were likely sequelae of escalation, some patients with religious beliefs agreed that limits on treatment would be preferable. Others felt that doctors are fallible, given the inherent uncertainty of clinical outcomes, and resisted doctors acting with ‘God power’, as one termed it, by withholding life-sustaining treatment in situations where patients or families believed survival was still possible.

‘We believe, this is our faith. We believe that you, as a doctor, has got to help as much as possible. But not when there is no solution, and keeping this machine. I don’t believe in this.’ —Female, aged 75–84, mild frailty, Arab ethnicity

The relationship between doctors and faith was complex. Some patients with religious beliefs perceived doctors to be an essential means by which God heals. Some felt that a person’s time and circumstances of death are predetermined, irrespective of the quality of medical decision-making or intervention. Patients also appeared able to reconcile acceptance of the limitations of medicine (and accordingly, a TEP with limitations on treatment escalation) with hope that they might be cured through faith.

‘[Faith plays] a very important role. In fact, I believe in God more than I believe in the medicine. He cured me not the medicine, the medicine’s the middle-man. The doctors are the middle-men, they deliver the medicine to you in order to cure you. But he gives you the green light, not the doctor, not the medicine. But if you give… If you believe in God you will be cured, it doesn’t matter what sort of illness you have. Even if you have got cancer you will be cured if you believe in him. And I do believe in him, that’s why I used the medicine as a middle-man, as a middle-item.’ —Male, aged 75–84, moderate frailty, any other ethnic group

**Table 5:** Selected illustrative quotations from clinicians’ and patients’ interviews. An extended version is in Appendix S5.

Theme	Quotes
**Clinicians theme 1:** **Decisions informed by a dynamic British medical way**	‘I’ve seen it with clinicians who were of the same culture as a family who one would have perceived to be asking for treatments which really are not indicated and won’t work. So I think the cultural clash then is between a particular cultural family and more the British medical way. But more and more we’re accepting if that’s the right word, of the cultural differences and we’re trying to support them.’ —*ICM Consultant* ‘And I think the other element that is quite important and is difficult sometimes to unpick are some of the cultural and religious elements to escalation. And I come from a cultural background where there isn’t a real strong belief in the sanctity of life. And I think most people from the cultural background I’m from would be very interested in quality of life over quantity of life. But that’s not necessarily true for all areas of society. And I think we sometimes struggle with that.’ —*ICM Consultant* ‘And so it’s a slightly personal position which I suppose you could argue is a medico-political-ethical position that I’ve taken that our job is to allow a nice death rather than to preserve life at all costs. And I suppose I’m possibly not burdened with a strong religious or cultural obligation to do absolutely everything to preserve life at any cost, maybe, I think.’ —*EM Consultant* ‘This comes down to, ultimately, personal opinion and your upbringing, your experience as a human, what you think is an acceptable quality of life, and culture. I went to a rehab hospital in Israel, and there were wards of patients in their 80s and 90s on ventilators, and it felt very uncomfortable and wrong. But for that culture it’s not. It’s what they want, and what they would feel is required, or for many people, anyway. So, yes, it’s culture-specific, I suppose, not just individuals.’ —*GIM Registrar* ‘Perhaps, my understanding is that if this man were in Italy, they probably would take him to ICU. So who knows what the right [thing] to do is.’ —*ICM Registrar* **Interviewer:** ‘When you’re involving the patient or their family in the discussion, what are you hoping to get from them?’ **Participant**: ‘So, I think a collateral. [. . .] So, an understanding of them as a person, their spiritual beliefs or culture they’ve come from and what’s important to them. So, definitely, that kind of thing is important.’—*GIM Registrar* ‘Maybe because I told you it’s my first year here in the UK. . . my point of view maybe a little bit different than what I’m seeing here. . .. He is 70, he’s an old patient, has a lot of multiple comorbidities, and very low functional capacity, even if his daily activities, can dress independently, but wife waits nearby. But still, for me, he’s a human being, fully conscious, maybe he wants to have another two, three years of living. So with this patient I will discuss and I will tell him that, if things got complicated, I will tell him that the treatment may be more painful, and may be more useless, but I will give him that choice.’*—ICM Registrar* ‘But we’re constantly living in fear that our colleagues either think we’re murderers, or nihilists, or over-investigators. And so we feel the need to fit in with the model around us, especially. . . And of course, we have our guidance, we have our GMC and our good practice, so we know the structures in which we need to work. But I think, sometimes, deviating from that can feel quite naked.’—*GIM Consultant*
**Clinicians theme 2:** **Cultural and clinical dissonance creates tension in TEP discussions**	‘The big differences happen I think when people have their religious or cultural beliefs. I’m trying to phrase it correctly, but. So strong that they can’t see the wood for the trees. And so they just get a line almost from their cultural backgrounds that one must do everything no matter what, without understanding the clinical picture as well. And there are times when I’m surprised when I start to explain to families about someone’s situation and they go, well, of course, you’re not going to do resuscitation and chest compressions and put them on a ventilator. She’s dying. So yes. But on the whole, I think if I do a good enough job of explaining why or why not I propose a particular treatment plan then families are accepting of that.’—*ICM Consultant* ‘And there comes then an interesting discussion with them about how much we can actually physically do to give them that, grant that wish, that want, and how much is simply beyond our capability. And that often comes up with a religious discussion with some people who are very, very embedded, either major religion or innate religious beliefs, that they have to take every single opportunity to prolong life regardless of what that life is, so our concepts of quality of life or autonomy don’t really come into it.’—*PC Consultant* ‘But I think it’s a lot of cultural, religious, and wider determinants. For example, in [a hospital], where I was working previously, mostly, people, they’re not white British. They’re usually South-East Asian or from the Muslim countries, and they just have very different perceptions of what doctors should be doing, and what the medicine should be doing. They don’t think about it as not doing anything as a good thing. That’s the opposite of what they think. So it’s very hard to change a cultural belief.’—*EM Registrar* ‘For example, when I’m speaking with Arabic family it’s hard to deliver bad news. I’m Arabic but I’m sorry to say that they don’t believe that there is a limitation for medicine. They think that people are like machines and whatever we do we should have a good effect. So delivering bad news, or that we will palliate or we will stop giving the medical treatment is not that easy. That’s why when I was working before in Arabic country we will just say giving more medication or intubation or CPR will cause more harm, but the decision will be them. And we will not insist in pushing them toward any decision. Because [. . .] they believe that everything we do should have an effect, like, yes.’—*ICM Registrar*
	**Interviewer**: ‘I was interested, a while back you were saying about, if you had your ten patients on the take, there’ll be X number where it’ll be really difficult, and there’ll be a considerable proportion where, actually, they’re really fine with having that escalation, the conversation. And I wonder, what do you think it is about those patients that makes them okay with having it?’ **Participant**: ‘I think there probably is a cultural element to it, and a religious element, [. . .] and an element of the extent of family involvement, and how much the patient wants the family to be involved. And I think, sometimes, there are situations where the patients, and even a medical relative, might say, we think that palliation might be more appropriate, but culturally. . . And the wider family don’t. They are actually easier conversations, because at least you can have the more nuanced discussion. As opposed to the people that say, do not play God, do not kill my mum, and they’re often more challenging.’—*GIM Consultant* ‘Yes, and culturally I think there are just some cultures where, regardless of the religion, say. . . People, I’ve noticed, from the Middle East, whether they’re Christian, Jewish or Muslim, will have certain opinions in terms of the family have the. . . As you were saying before, have the strong decision-making ability, and the patients will often defer to the family. And that the idea of death is still. . . And sometimes people will say, actually, I know but I need to advocate for my mum, and therefore I need to keep arguing with you for full resus because that, for me, is advocating for my mum.’—*GIM Consultant* **Interviewer**: ‘So I think many of us are just about all right with saying, this patient has a view which is different from mine, but I will respect it because I must. But when the patient’s view is that they will defer to their family, and then the family say they want something that is different from what we would want, I wonder if that, does that then take it a step too far? Because still, in theory, that is what is most important to the patient, but I think I feel we struggle from that.’ **Participant**: ‘I agree, and I think that’s so far from our medical upbringing, and from what we believe is the right thing. Where, of course, we have to follow the GMC, which is the patient is the most important person to you. I agree, I find it very jarring.’—*GIM Consultant* ‘The problem is if there is a chance that you’d survive, even if it’s a much-reduced quality of life, and that’s what the patient would want, I find it difficult to not offer that. Because why wouldn’t we offer that? What is it that we’re losing out on by not offering that? If they feel that they would want it, it’s resources. But I’m not sure we’re the people who should be limiting resources. Because if resources should be limited and inform those decisions, there ought to be clear guidance how we do that. So if you think there is some chance of recovery, and the family say, we would be. . . Or the patient, more importantly, says, yes, I would be happy to be alive at any cost. Then I think it’s difficult not to offer it.’—*ICM Consultant, in the context of patients asking for treatment escalation on religious grounds* ‘If you bring someone who they’re going to ventilate for the next three months and then die, that doesn’t keep morale very good. And so that also, along with cost, is important to take into account when you’re making these kind of decisions. But I think, for me, on a personal level, with my religious belief, I make sure that doesn’t interfere in my decision making. I know those are important factors, but I don’t let them. . . However, there are important things to also consider.’—*ICM Consultant*
**Clinicians theme 3:** **Convincing and compromising: negotiating a culturally acceptable decision**	‘I would try and challenge some of it, and talk to him about life being sacred and try and find out if he’s. . . It sounds like he’s religious. Try and offer some religious support. Try and ask him if he was worried about dying. I think you try and explain to the patient that an attempt to chest compressions is not what resuscitation is. Either you do it properly and you fully resuscitate someone or you don’t, and that it’s a medical decision to treatment, like any other treatment. But we want him to understand the rationale on which the treatment is based or the decision is based.’—*GIM Consultant* ‘I think we generally do have a gut feeling one way or the other about these things. But ultimately I think we leave it, not leave it up to the patient, but achieve a compromise as best as possible, if there’s a strong conflict. But I don’t think we necessarily change our minds about it.’—*GIM Registrar* ‘I think it’s also to get a feel what the family think. Because say that I were to still exercise my decision, I’d want to know what the family would feel at the end of it all. If the family also hold really strong opinions like him, then I think it goes back to the point I was saying earlier about not wanting to break down that whole doctor patient relationship for the long run. So, then I think we may have to end up achieving a halfway house. But I think if we’re able to explain to the family, and they understand, then they may be able to explain to the patient what exactly we’re trying to say. And then it comes from a place where it’s someone who cares for them telling them that. So, I think the aim is still to convince the patient that this is not a good idea by involving more people saying the same thing.’—*GIM Registrar, in response to a question about involving family in decision-making for a capacitous patient requesting further escalation on religious grounds* ‘I push back a little and I would say, can we speak to a religious person like an imam or, I don’t know, a chaplain or that sort of thing. I think if it were just me and him, then I think that would be a conversation that we can have, leave it between us, there’s no need to escalate. But then if the family are going to make the hospital and the junior doctors and the staff nurse’s lives very difficult and there’s a chance that it’s going to get out of hand, unfortunately it isn’t just the relationship between the patient, it becomes the whole hospital, NHS reputation, then you’d have to escalate to someone, the medical director or whatever’ . . .. ‘But I would say no. If I was the medical director, I would say, I’m sorry, this is not something we would do.’—*GIM Consultant*
	**Interviewer:** ‘Would you ever say, we’ll leave you for full escalation, we’ll leave you for everything, because this is what you want, or do you think it just really doesn’t sit well?’ **Participant**: ‘It doesn’t sit with me, no. I would get other people on board to support my decision, but I wouldn’t do it.’ **Interviewer**: ‘What makes you feel that way?’ **Participant**: ‘Because I would be harming the patient and I would be doing something that doesn’t sit well with me, doesn’t sit comfortably.’—*GIM Consultant, in the context of conflict with a patient requesting further escalation on religious grounds* ‘So, I think we for want of a better word, the more professional use of the word, we respect [cultural differences] and therefore we try and support families when we feel that somebody is in a palliative care situation. So, if somebody’s come in intubated yet they’ve had a catastrophic brain injury and they’re a frail 88-year old but for some reason, they’ve been intubated and if the family can’t accept that then we give them time. But you just say to them, I’m not going to increase the organ support. There’s a chance that the organs could stop. There has to be a line where you go, I’m not going to do this, I’m not going to do that. But if something has already been started then you give time and support. I often say we’ll probably forget this particular patient and their family because we do this so much but they’ll always remember and so, therefore, you end up changing the way that you are. You’re supporting the patient to a degree but actually, you’re supporting the family in the end-of-life process. [. . .] But yes, I think the way that we have moved from, I don’t know, decades ago is that we will offer some support in order to give time for families to accept. And that’s usually what people need is time.’—*ICM Consultant*
**Patients’ theme 1:** **A relational decision made within a family network**	
Subtheme 1: Making a personal decision is an interpersonal process	‘But sometimes they don’t. Their brain, this capability of thinking is gone because of the medications and because of their illnesses and everything. I think this is when, I think, in our custom, in our Indian culture, generally they are the sons. They try to talk to the sisters and everyone, and they try and make a decision.’—*Female, aged 75–84, mild frailty, Indian ethnicity* ‘They can talk to the patient, depending on how acceptable the patient is, how strong and willing the patient is. If you notice that the patient is not very strong to accept the proposals, then it’s better to talk to the family. In most cases, I’ve seen that patients who are deteriorating, doctors talk to the family. Yes, this is what we suspect, this is what is going to be happening, so this is what we are prepared to do. What do you say? Then the family, the key person in the family, they discuss it with the patient. This is somebody the patient has confidence in, that is close to the patient, would talk to her or him or would agree that the doctors communicate directly with the patient. They would say, oh, no, you talk to her, she would rather understand when you talk than me. . .’—*Female, aged 65–74, no frailty, African ethnicity*
Subtheme 2: Familial caregiving as a cultural ideal	‘You have come to the Intensive Care the whole week, it’s going to two weeks, your condition remains the same. The family is always important in such cases. Even the patient is always important in such cases, as long as the patient can take a decision whether to continue to stay in the Intensive Care or to go out to the ward. Because some people feel better when there are people around them. So when you restrict that, they don’t improve. They themselves wonder, oh, what is wrong with me? But I’m not improving. I’m getting all the medication, but I’m not improving. Maybe I’m missing something. Family. People coming to visit.’—*Female, aged 65–74, no frailty, African ethnicity* Husband: ‘Family encouragement is very important, that motivates you to live.’ Wife: ‘Yes, but not carers.’—*Husband: Male, aged 75–84, moderate frailty, Any other ethnic group. Wife: Female, aged 75–84, mild frailty, Any other ethnic group* ‘It depends how much family support he has at the back. Nowadays, nobody cares. If somebody asks, they might give him one or two and then they get fed up if you are in the bed. Say he’s living with his son and daughter or his wife, whatever, they might respond to his request if there are a few. They cannot do it for all of their life. They will get fed up and then they try to dump him in the carer’s home.’—*Male, aged 65–74 years, moderate frailty, Indian ethnicity* ‘Yes. And you know and I know, when the carer comes, and I’m telling you from my own experience, they are lovely people, but they can only go so far. They can only go by the books. And otherwise, they will not. So we actually did more for our parents than the carer came, but my father will not allow the carer to touch him. So it is such a vast subject, how to give the patient the best.’—*Female, aged 75–84, mild frailty, Indian ethnicity*
**Patients theme 2:** **Expectations built on experiences in different contexts**	‘Yes, I can do both. I still trust the doctor. I trust what they’re doing and that is why, in the Western World, we have this opportunity of the expert helping you, prolonging our lives [. . .]. And a number of people I know, they’ve died, third world country or these things, all because of the health we get here.’—*Male, aged 75–84, no frailty, African ethnicity* ‘Even in the Philippines general hospital, I worked in the charity ward. You can’t imagine people sometimes. They don’t have money to buy. And you know the supply, we are the government hospital. We are not like NHS. You have everything. But still we give chance.’ ‘But you have to do your best to let them survive. And sometimes they survive. Not only sometimes, most of them survive.’—*Male, aged 65–74, no frailty, any other Asian background [previous nurse in home country]*
**Patients theme 3:** **Expert decision, divine outcome**	‘Well, I think it’s right because they’re specialist and expert in their own field. If I get to that stage and they give me, I would take it. And as I’ve said, as a Christian, I have alternative solution, an alternative, I would take it because nobody wants to die really. I go to that treatment. If it improve my health, I happy. If it doesn’t, turn to my spiritual belief. I will use it. I won’t turn anybody down when you get to that stage.’—*Male, aged 75–84, no frailty, African ethnicity* **Interviewer**: ‘Do you mean that lots of Jewish people might think it’s an important conversation, or did I misunderstand?’ **Participant**: ‘No, I can’t think that some people would ignore advice from doctors. We treat them as reliable, sensible people.’—*Female, aged 85+, moderate frailty, Any other White background* ‘As far as my religion is concerned, they know we have to go one day. It doesn’t matter if you live 60 years or 100 years. One day is decided. It doesn’t matter even if you are in hospital. It doesn’t matter if you see a good consultant or a very famous one, when the time comes it’s up. Time to go. Nobody will save you.’—*Male, aged 65–74 years, moderate frailty, Indian ethnicity* ‘If He wants to take me, He will take me. If he wants to cure me, He will cure me. He will cure me through you, through the doctor.’—*Female, aged 75–84, mild frailty, Arab ethnicity* **Interviewer**: ‘What would be your perspective from a faith point of view, on that? Is that worth doing, or not?’ **Participant**: ‘No [. . .] If you are certain, as a doctor, that if you try this machine, and I will still be a vegetable, or not conscious at all, no, you shouldn’t do it, no.’—*Female, aged 75–84, mild frailty, Arab ethnicity* ‘He should have the right to live his life. The doctors should not be the God power to decide this person should die and this person should live.’—*Male, aged 65–74 years, moderate frailty, Indian ethnicity* “Let them decide. Because keeping the person, I think, is just. . . Although we pray and pray, and that’s the last resort, the prayer. That’s what most of us believe in, that we will pray, and then we’ll come back to life. But if you’re coming back to life, and your life is not worth it, so I don’t think you should come back to life just to give your family more stress, more agony, in seeing you in that condition.”—*Female, aged 65–74, no frailty, African ethnicity* ‘You can be young. It can be old age. You have to leave this place. But what I always pray for myself is that Allah, I mean, God should take me without being dependent on others for my day-to-day living.’*—Male, aged 65–74, moderate frailty, Indian ethnicity* ‘It’s not a religious thing, but it is something to do with your faith. We say, leave it to Allah, leave it to God, He will take care of me. That’s what we believe. So this is how I will think of it.’—*Female, aged 75–84, mild frailty, Arab ethnicity* ‘We live for our beliefs, and faith, as much as we live for our children, and family, and friends. You have to have a belief, if you don’t have a belief you have nothing.’—*Female, aged 75–84, mild frailty, Any other ethnic group*

## Discussion

This secondary analysis presents exploratory insights into patients’ and clinicians’ perspectives on cultural and religious influences in shared decision-making for treatment escalation planning. First, the research considers clinicians’ attitudes towards their own and patients’ cultural or religious approaches to TEP decision-making. Clinicians were informed by professional cultural norms based on notions of clinically beneficial treatment, individual autonomy and resources; these could feel challenged by other belief systems. Next, we explore how culture and religion influenced patients’ thinking around TEPs: patients were motivated by faith, family and previous healthcare experience. Through outlining these different frameworks, we can start to understand domains in which there may be potential challenges in TEP, offering a foundation for more nuanced and culturally attuned approaches to decision-making in acute care.

Clinicians described a strong commitment to reaching what they considered the ‘right’ decision, guided by a collective understanding of ethical and professional norms in a pervasive, though difficult to define, British medical culture. Accounts from clinicians with experience in other healthcare systems further highlighted that decision-making instincts are shaped by cultural context. Clinical decisions, including those around TEP, are frequently shaped by such norms [31–34]. One example is the concept of ‘mindlines’, where clinicians subconsciously draw on tacit, collectively held knowledge to guide decision-making [35, 36]. These normative frameworks can be valuable for complex decisions and consistent clinician communication [37] but can be difficult to deviate from and may not accommodate case-specific nuances [31]. Consistent with previous studies, [38–40] clinicians in this study described moral distress when it was difficult to reconcile clinically-informed decision-making, resource consciousness and established decision-making norms with a commitment to patient-centred care and maintaining the relationship with the patient and family through accommodating cultural or religious priorities. Medical decisions were often communicated with strong conviction and persuasive intent, and, consistent with other studies [38, 41–43], this contributed to tensions when those decisions were challenged. As societal and professional cultural diversity shift over time in a changing geopolitical landscape, it is likely that the nature of the British medical culture and how this interacts with patient views will continue to evolve [1, 44].

Differences in ideas about autonomy influenced beliefs around decision-making. Patients from some cultures emphasised the role of family as a unit of agency, preferring collective decision-making and familial care provision. Some clinicians found this approach challenging, as British societal norms and medical guidelines prioritise the patient as an individually autonomous decision-maker [11, 12, 19, 21]; clinicians recalled difficulties navigating family dynamics and understanding how these influenced expressed patient preferences. Other studies in Western healthcare contexts have similarly observed differences in the locus of autonomy between patients and clinicians, particularly in relation to end-of-life care planning [9, 45–50]. This poses questions about the applicability of individualistic notions of autonomy for patients from wider ethnic and cultural backgrounds, especially in decisions with important emotional and practical implications for family members. A relational approach to autonomy [51, 52], acknowledging that patients may prefer to make decisions with reference to others, may be more congruent with some belief systems. Although this approach is a central principle in the care of older people, it is rarely formalised in UK policy or clinical guidance. In practice, this may involve honouring patients’ choices to delegate decision-making to relatives. This, however, may feel uncomfortable to clinicians [52], especially if relatives request treatments that clinicians consider harmful, or if there are consequences for unequal resource allocation [42, 51]. These challenges are difficult to resolve.

Differences in expectations for treatment were a key source of tension in TEP discussions. As previously [53], some patients’ expectations in our study were influenced by exposure to other healthcare systems. Clinicians sought a shared understanding about TEPs to align expectations but remarked that this was not always successful. In some settings, treatment escalation to the maximum level available is the standard approach, there is little exposure to palliative care or resource limitations mean that available treatment is sometimes extremely modest, and so the concept of limiting effort and focusing solely on symptom control appears illogical. In contrast, Western healthcare environments are perceived by some immigrant communities to have almost infinite scope to improve health due to abundant medical expertise and equipment [54]. Establishing a constructive relationship early in the illness trajectory and conducting iterative, values-based conversations may help to reduce conflict during moments of crisis [9, 42, 53, 54], but this may not always be practical in some acute settings.

Clinicians frequently anticipated conflict with patients and families from certain minority backgrounds, expecting them to prioritise life-prolongation over quality of life and to demand treatments deemed clinically inappropriate. However, patient participants in this study and others expressed a spectrum of perspectives shaped by varied individual interpretations of religious and cultural tenets [9, 53], which were central to their understanding of acceptable outcomes and informed their definitions of good care [54]. Our participants’ acceptance of treatment ceilings depended on how they integrated the illness trajectory, the perceived purpose of medical intervention and its impact on their community and, for religious patients, their personal understanding of Divine Will. Consequently, while some patients felt a duty to pursue maximal intervention, others considered treatment limitations compatible with their religion or culture. Previous research has suggested that conflict with patients from different backgrounds may not be attributable solely to cultural factors but rather their friction with triggering structural factors in healthcare settings operating on fundamentally different principles [54]. Therefore, asymmetry between clinicians’ and patients’ recollections of culture-based conflict in this study may reflect clinicians drawing upon personal experiences of challenging clinical scenarios which triggered conflict, while patients spoke more hypothetically about the acute medical setting. Nonetheless, it is important to remark that patients’ interpretations of the intersection between faith and TEP were complex and highly individual, precluding any single, collective interpretation.

## Implications

Cultural competency training, which aims to increase clinicians’ knowledge and understanding of issues around culture and health [55], has been proposed as a means to address clinicians’ confidence and improve care for patients from diverse backgrounds [42, 56]. However, others argue its efficacy is limited without wider structural changes to healthcare operations [43, 54]. This study provides a nuanced understanding of how patients and clinicians view religion and culture in TEP decision-making, enabling us to make the following recommendations.

For clinicians, reflections from this analysis can help build understanding on the wider cultural context and values shaping patients’ perspectives. This insight may facilitate more amicable communication or at least help clinicians to manage the emotional burden of navigating conflicting priorities. While there is significant heterogeneity within cultural and religious groups, and an individualised approach is paramount [9, 57, 58], reflections on the socially constructed notion of a ‘British medical culture’ and how this interacts with other perspectives may be formative.

For policymakers, we have highlighted autonomy as a domain viewed differently by clinicians and some patients. Individual patient autonomy is currently prioritised in decision-making guidelines [11, 12, 19, 21]. For patients who value familial involvement, person-centred care may be better supported by integrating a relational approach to autonomy, validating opinions developed from a collective family unit [51, 52, 59]. Prompts to familial discussions, where relevant and welcomed by patients, could be integrated in standardised TEP documentation [51]. Guidance from regulatory bodies may be helpful when balancing culturally informed treatment requests with resource constraints. Systems-level changes allowing more flexibility on the timing and length of these conversations may reduce associated tensions, but may be challenging to integrate in acute care workflows navigating significant clinical pressures.

## Limitations

This exploratory, opportunistic secondary analysis utilised data from two primary studies that were not designed to explore cultural and religious influences in TEP specifically. This contributes to the following limitations:

First, as this was a preliminary exploration of an under-researched area, we prioritised breadth and openness to avoid imposing any assumptions. We have not specified a definition for ‘culture’, recognising that it is an inexact and debated term [60], and chose instead to respect participants’ own interpretations. This approach may have missed more subliminal accounts of cultural and religious influences that were not explicitly labelled as such by participants, due to participants’ interpretations, interactions between participants, interviewer and analyst positionality and our own biases; for example, we observed that patients from White British backgrounds are less represented. However, by including self-reported accounts of how culture and religion influence TEP decision-making, we have captured a wide range of experiences without excluding participants from any background or belief system.

Second, sampling was not tailored to the research question. We did not formally recruit participants to represent specific belief systems and have not captured a full diversity of cultural perspectives. Also, patients who discussed relevant ideas were generally less frail (CFS none to moderate); frailty may influence treatment escalation preferences and/or views on shared decision-making. Future research would look to purposively sample across cultural and religious backgrounds and frailty levels to explore how healthcare experiences intersect with different cultures’ perspectives and belief systems in shaping care preferences. It would also be important to explore more directly how clinicians’ cultural and religious backgrounds influence TEP decision-making.

Third, the topic guide was not focussed on cultural or religious perspectives. Consequently, ideas were expressed in different contexts by patients and clinicians; clinicians’ prompts and reflections were sometimes more focussed on conflict scenarios. The depth of probing was also variable, potentially limiting interpretations from participants whose ideas were not more intentionally explored. Nonetheless, that so much data on cultural factors was volunteered spontaneously implies their significance.

Limitations specific to the primary studies are reported separately [22, 23].

## Conclusion

This qualitative secondary analysis explored cultural and religious influences on shared decision-making in treatment escalation planning from clinician and patient perspectives. A British medical culture guiding TEP decision-making is juxtaposed with diverse patient perspectives. Mismatch between clinicians’ and patients’ cultural and religious priorities could result in conflict and moral distress and be related to different interpretations of individual autonomy, treatment limitations and the role of clinicians in highly existential decisions. While clinicians’ and patients’ notions of a good TEP decision may be different, each is valid and adaptable. Our findings provide insight for clinicians conducting TEP conversations. We suggest that more nuanced, flexible guidelines incorporating diverse perspectives would support a more culturally sensitive framework for decision-making. A challenge for future research is to consider how to formulate guidelines integrating clinical priorities, cultural or religious needs and equitable resource use for high-stakes decisions in acute settings.

## Supplementary Material

Supplementary_materials_afaf357

## Data Availability

Select anonymised interview data may be shared upon reasonable request to the corresponding author.

## References

[1] Cuibus MV ; Migrants in the UK: An Overview. The Migration Observatory, 2024.

[2] Office for National Statistics . *Ethnic group, England and Wales: Census 2021*. https://www.ons.gov.uk/peoplepopulationandcommunity/culturalidentity/ethnicity/bulletins/ethnicgroupenglandandwales/census2021 2022, (date accessed 25/11/2024)

[3] Office for National Statistics . *Religion, England and Wales: Census 2021*. https://www.ons.gov.uk/peoplepopulationandcommunity/culturalidentity/religion/bulletins/religionenglandandwales/census2021 2022, (date accessed 25/11/2024)

[4] Cain CL, Surbone A, Elk R et al. Culture and palliative care: preferences, communication, meaning, and mutual decision making. J Pain Symptom Manag 2018;55:1408–19. 10.1016/j.jpainsymman.2018.01.007. 29366913

[5] Woll ML, Hinshaw DB, Pawlik TM. Spirituality and religion in the Care of Surgical Oncology Patients with life-threatening or advanced illnesses. Ann Surg Oncol 2008;15:3048–57. 10.1245/s10434-008-0130-9. 18773242

[6] Kagawa-Singer M, Dressler W, George S et al. Culture: the missing link in health research. Soc Sci Med 2016;170:237–46. 10.1016/j.socscimed.2016.07.015. 27542574

[7] Kagawa-Singer M, Dressler W, George S et al. *The Cultural Framework for Health*. Washington, DC: National Institute for Health, 2015.

[8] Choudry M, Latif A, Warburton KG. An overview of the spiritual importances of end-of-life care among the five major faiths of the United Kingdom. Clin Med 2018;18:23–31. 10.7861/clinmedicine.18-1-23. PMC6330909 29436435

[9] Islam Z, Pollock K, Patterson A et al. Thinking ahead about medical treatments in advanced illness: a qualitative study of barriers and enablers in end-of-life care planning with patients and families from ethnically diverse backgrounds. Health Soc Care Deliv Res 2023;11:1–135. 10.3310/JVFW4781. 37464868

[10] Public Health England . *Faith at end of life: public health approach resource for professionals*. https://www.gov.uk/government/publications/faith-at-end-of-life-public-health-approach-resource-for-professionals 2016, (date accessed 26/11/2024)

[11] General Medical Council . *Treatment and Care towards the End of Life: Good Practice in Decision Making*. London: General Medical Council, 2010.

[12] Department of Health and Social Care . *End of Life Care Strategy: promoting high quality care for adults at the end of their life*. https://www.gov.uk/government/publications/end-of-life-care-strategy-promoting-high-quality-care-for-adults-at-the-end-of-their-life 2008, (date accessed 26 November)

[13] The Health Foundation . *Trends in the number of English NHS hospital admissions, 2006 to 2016*. https://www.health.org.uk/chart/chart-trends-in-the-number-of-english-nhs-hospital-admissions-2006-to-2016 2018, (date accessed 25 November

[14] Faitna P, Bottle A, Klaber B et al. Has multimorbidity and frailty in adult hospital admissions changed over the last 15 years? A retrospective study of 107 million admissions in England. BMC Med 2024;22:369. 10.1186/s12916-024-03572-z. 39256751 PMC11389502

[15] Salive ME . Multimorbidity in older adults. Epidemiol Rev 2013;35:75–83. 10.1093/epirev/mxs009. 23372025

[16] Marengoni A, Angleman S, Melis R et al. Aging with multimorbidity: a systematic review of the literature. Ageing Res Rev 2011;10:430–9. 10.1016/j.arr.2011.03.003. 21402176

[17] Psirides A, Tripp DG, Pegg TJ. Rethinking resuscitation: moving the goals. N Z Med J 2021;134:83–8. 34482392

[18] Hawkes CA, Fritz Z, Deas G et al. Development of the recommended summary plan for emergency care and treatment (ReSPECT). Resuscitation 2020;148:98–107. 10.1016/j.resuscitation.2020.01.003. 31945422

[19] British Medical Association, Resuscitation Council UK, Royal College of Nursing . *Decisions relating to cardiopulmonary resuscitation.* https://www.resus.org.uk/sites/default/files/2020-05/20160123%20Decisions%20Relating%20to%20CPR%20-%202016.pdf 2016, (date accessed 25/11/2024) 10.1136/jme.27.5.310 PMC1733441 11579186

[20] National Institute for health and care excellence . *Shared decision making (NG197)*. www.nice.org.uk/guidance/ng197 2021, (date accessed 26/11/2024) 34339147

[21] General Medical Council ; Decision making and consent. London: General Medical Council, 2020.

[22] Warner BE, Wells M, Vindrola-Padros C et al. Shared decision-making with older people on TReatment escalation planning for acute deterioration in the emergency medical setting: a qualitative study of clinicians’ perspectives (STREAMS-C). Age Ageing 2024;53:afae204. 10.1093/ageing/afae204. 39323400 PMC11424886

[23] Warner BE, Wells M, Vindrola C et al. Shared decision making with older people on treatment escalation planning for acute deterioration in the emergency medical setting: a UK-based qualitative study of patient perspectives (STREAMS-P). Lancet Healthy Longev 2025;6:100689. 10.1016/j.lanhl.2025.100689. 40058387

[24] Rockwood K, Song X, MacKnight C et al. A global clinical measure of fitness and frailty in elderly people. CMAJ 2005;173:489–95. 10.1503/cmaj.050051. 16129869 PMC1188185

[25] gov.uk . *Regional ethnic diversity 2011 census*. https://www.ethnicity-facts-figures.service.gov.uk/uk-population-by-ethnicity/national-and-regional-populations/regional-ethnic-diversity/latest 2018, (date accessed 02/01/2023)

[26] Braun V, Clarke V. *Thematic Analysis: A Practical Guide*. London: SAGE, 2022.

[27] Warner BE . Clinical colleague and clinical academic: a Physician’s autoethnographical reflexive account of opportunities and challenges as an ‘insider’ researcher. Int J Qual Methods 2024;23:16094069241256278. 10.1177/16094069241256278.

[28] Braun V, Clarke V. Reporting guidelines for qualitative research: a values-based approach. Qual Res Psychol 2025;22:399–438. 10.1080/14780887.2024.2382244.

[29] Long-Sutehall T, Sque M, Addington-Hall J. Secondary analysis of qualitative data: a valuable method for exploring sensitive issues with an elusive population? J Res Nurs 2011;16:335–44. 10.1177/1744987110381553.

[30] Gladstone BM, Volpe T, Boydell KM. Issues encountered in a qualitative secondary analysis of help-seeking in the Prodrome to psychosis. J Behav Health Serv Res 2007;34:431–42. 10.1007/s11414-007-9079-x. 17694437

[31] Reader TW, Geetha R, Brett SJ. Impossible decision? An investigation of risk trade-offs in the intensive care unit. Ergonomics 2018;61:122–33. 10.1080/00140139.2017.1301573. 28300480

[32] Dixon-Woods M, Baker R, Charles K et al. Culture and behaviour in the English National Health Service: overview of lessons from a large multimethod study. BMJ Qual Saf 2014;23:106–15. 10.1136/bmjqs-2013-001947. PMC3913222 24019507

[33] Curtis JR, Barnato AE. Variability in decisions to limit life-sustaining treatments: is it all about the physician? Chest 2014;146:532–4. 10.1378/chest.14-0636. 25180716

[34] Barnato AE, Tate JA, Rodriguez KL et al. Norms of decision making in the ICU: a case study of two academic medical centers at the extremes of end-of-life treatment intensity. Intensive Care Med 2012;38:1886–96. 10.1007/s00134-012-2661-6. 22940755 PMC3684418

[35] Gabbay J, le May A. Evidence based guidelines or collectively constructed "mindlines?" ethnographic study of knowledge management in primary care. BMJ 2004;329:1013. 10.1136/bmj.329.7473.1013. 15514347 PMC524553

[36] Wieringa S, Greenhalgh T. 10 years of mindlines: a systematic review and commentary. Implement Sci 2015;10:45. 10.1186/s13012-015-0229-x. 25890280 PMC4399748

[37] Griffiths F, Svantesson M, Bassford C et al. Decision-making around admission to intensive care in the UK pre-COVID-19: a multicentre ethnographic study. Anaesthesia 2021;76:489–99. 10.1111/anae.15272. 33141939

[38] Eli K, Hawkes CA, Ochieng C et al. Why, when and how do secondary-care clinicians have emergency care and treatment planning conversations? Qualitative findings from the ReSPECT evaluation study. Resuscitation 2021;162:343–50. 10.1016/j.resuscitation.2021.01.013. 33482270

[39] Dzeng E . Habermasian communication pathologies in do-not-resuscitate discussions at the end of life: manipulation as an unintended consequence of an ideology of patient autonomy. Sociol Health Illn 2019;41:325–42. 10.1111/1467-9566.12825. 30460704 PMC6359964

[40] Miller S, Dorman S. Resuscitation decisions for patients dying in the community: a qualitative interview study of general practitioner perspectives. Palliat Med 2014;28:1053–61. 10.1177/0269216314531521. 24815004

[41] Eli K, Ochieng C, Hawkes C et al. Secondary care consultant clinicians' experiences of conducting emergency care and treatment planning conversations in England: an interview-based analysis. BMJ Open 2020;10:e031633. 10.1136/bmjopen-2019-031633. PMC7044868 31964663

[42] Islam Z, Taylor L, Faull C. Thinking ahead in advanced illness: exploring clinicians’ perspectives on discussing resuscitation with patients and families from ethnic minority communities. Future Healthc J 2021;8:e619–24. 10.7861/fhj.2021-0012. 34888453 PMC8651317

[43] Kai J, Beavan J, Faull C et al. Professional uncertainty and disempowerment responding to ethnic diversity in health care: a qualitative study. PLoS Med 2007;4:e323. 10.1371/journal.pmed.0040323. 18001148 PMC2071935

[44] NHS England . *NHS Workforce Race Equality Standard*. https://www.england.nhs.uk/long-read/nhs-workforce-race-equality-standard-wres2022-data-analysis-report-for-nhs-trusts/ 2023, (date accessed 28/11/2024)

[45] Brown EA, Bekker HL, Davison SN et al. Supportive care: communication strategies to improve cultural competence in shared decision making. Clin J Am Soc Nephrol 2016;11:1902–8. 10.2215/CJN.13661215. 27510456 PMC5053803

[46] Bellamy G, Gott M. What are the priorities for developing culturally appropriate palliative and end-of-life care for older people? The views of healthcare staff working in New Zealand. Health Soc Care Community 2013;21:26–34. 10.1111/j.1365-2524.2012.01083.x. 22812427

[47] Calanzani N, Koffman J, Higginson IJ. Palliative and End of Life Care for Black, Asian and Minority Ethnic Groups in the UK: Demographic profile and the current stateof palliative and end of life care provision, 2013.

[48] Nguyen N, Zivkovic T, de Haas R et al. Problematizing "planning ahead": a cross-cultural analysis of Vietnamese health and community workers' perspectives on advance care directives. Qual Health Res 2021;31:2304–16. 10.1177/10497323211023453. 34369232

[49] Zivkovic T . Forecasting and foreclosing futures: the temporal dissonance of advance care directives. Soc Sci Med 2018;215:16–22. 10.1016/j.socscimed.2018.08.035. 30196148

[50] Venkatasalu MR, Arthur A, Seymour J. Talking about end-of-life care: the perspectives of older south Asians living in East London. J Res Nurs 2013;18:394–406. 10.1177/1744987113490712.

[51] Gomez-Virseda C, de Maeseneer Y, Gastmans C. Relational autonomy: what does it mean and how is it used in end-of-life care? A systematic review of argument-based ethics literature. BMC Med Ethics 2019;20:76. 10.1186/s12910-019-0417-3. 31655573 PMC6815421

[52] Gilbar R, Miola J. One size fits all? On patient autonomy, medical decision-making, and the impact of culture. Med Law Rev 2015;23:375–99. 10.1093/medlaw/fwu032. 25520516

[53] Kirby E, Lwin Z, Kenny K et al. “It doesn’t exist…”: negotiating palliative care from a culturally and linguistically diverse patient and caregiver perspective. BMC Palliat Care 2018;17:90. 10.1186/s12904-018-0343-z. 29966521 PMC6027583

[54] Van Keer R-L, Deschepper R, Francke AL et al. Conflicts between healthcare professionals and families of a multi-ethnic patient population during critical care: an ethnographic study. Crit Care 2015;19:441. 10.1186/s13054-015-1158-4. 26694072 PMC4699338

[55] NHS England . *Cultural Competence and Cultural Safety*. https://www.e-lfh.org.uk/programmes/cultural-competence/ 2025, (date accessed 31/05/2025)

[56] Renzaho AMN, Romios P, Crock C et al. The effectiveness of cultural competence programs in ethnic minority patient-centered health care—a systematic review of the literature. Int J Qual Health Care 2013;25:261–9. 10.1093/intqhc/mzt006. 23343990

[57] Degrie L, Gastmans C, Mahieu L et al. How do ethnic minority patients experience the intercultural care encounter in hospitals? A systematic review of qualitative research. BMC Med Ethics 2017;18:2. 10.1186/s12910-016-0163-8. 28103849 PMC5244561

[58] Diver F, Molassiotis A, Weeks L. The palliative care needs of ethnic minority patients attending a day-care Centre: a qualitative study. Int J Palliat Nurs 2003;9:389–96. 10.12968/ijpn.2003.9.9.11766. 14593275

[59] Giusti A, Nkhoma K, Petrus R et al. The empirical evidence underpinning the concept and practice of person-centred care for serious illness: a systematic review. BMJ Glob Health 2020;5. 10.1136/bmjgh-2020-003330. PMC7733074 33303515

[60] Spillman L . *What Is Cultural Sociology?* 1-21.United Kingdom: Polity Press, 2020.

