# Supplementary material for: Cultural and religious influences on shared decision-making in treatment escalation planning with older adults in the acute setting: a qualitative secondary analysis of interview studies seeking clinician and patient perspectives
Source: Age Ageing. 2025 Dec 17;54(12):afaf357. doi: 10.1093/ageing/afaf357 (PMC12708056; doi:10.1093/ageing/afaf357)
Supplement: Supplementary_materials_afaf357 [file supplementary_materials_afaf357.docx]

**Cultural and religious influences on shared decision-making in treatment escalation planning with older adults in the acute setting: a qualitative secondary analysis of interview studies seeking clinician and patient perspectives**

**Supplementary materials**

**Contents**

1. Appendix 1 – Clinician study topic guide
2. Appendix 2 – Patient study topic guide
3. Appendix 3 – Data analysis
4. Appendix 4 – Reflexivity
5. Appendix 5 – Extended table of illustrative quotations
6. References for supplementary materials

**Appendix 1**

**Clinician study topic guide (1)**

Participants were asked to describe their approach to TEP, invited to discuss three vignettes desciribing a hypothetical patient, and finally directly asked their views on SDM in TEP. The vignettes were developed by clinical members of the primary research team, and described a capacitous patient with multiple interacting comorbidities being admitted under the acute medical team for a urinary tract infection, but not acutely deteriorating. Across the scenarios, age, severity of comorbidity and functional dependence increased. *Clarifying questions added during the first four interviews are shown in italics.*

**INITIAL TOPIC GUIDE**

- Explore clinicians’ approach to treatment escalation planning conversations with a focus on the perceived role of shared decision making
- Consider whether the treatment escalation planning conversation or decision vary with regards to shared decision making depending on patient clinical factors

Thank you for agreeing to participate in this study. Today I’d like to find out how you think about decision making around treatment escalation planning for patients admitted to hospital on the acute medical take.

I have some scenarios to help set the scene and some questions to make sure I’ve covered everything but really this should just be a conversation.

This should take no more than one hour. I will be recording our conversation so that I’m free to listen to you.

Everything you say will be confidential and anonymised for the study. Do you have any questions? Are you happy to proceed?

1. What is your understanding of Treatment Escalation Planning (/respect form/ceiling of care decisions)?

- What does it mean to you (in the acute medical setting)?
- Is it useful?
- When are you involved in TEP, how do you see your role?
- *Has your approach changed over time, if so why?*

1. How would you approach Treatment Escalation Planning for each of these patients?

You have been given three scenarios. Please read them now.

I’d like you to imagine you are making a treatment escalation plan for each of these patients.

- Can you talk me through step by step how you would approach it?
  - *what is an ‘acceptable’ outcome, how do you decide a threshold, how bad is too bad?*
  - *how/why does your view change across the three scenarios?*
  - *Is there a ‘right’ decision?*
  - *what is ‘futility’?*
  - *would it make a difference if there were unlimited resources?*
- What sort of conversation would you have with the patient?
  - *do you ever talk about shared goals of care?*
- Are you more likely to think/talk about TEP for some patients than others?

1. Involving patients and families in Treatment Escalation Plans in general?

- What do you think about involving them?
  - *where are you on a spectrum from giving information and leaving patients to make a decision, recommending a decision, communicating your decision*
- How/when would you involve them – can you talk me through?
- Would you explain the risks, benefits, alternatives of your proposed TEP?
- What if there is disagreement?
  - *how much is litigation a concern?*
- What would make you think the conversation had gone well?
- What do you think about shared decision making in treatment escalation planning?
- *What is the most important thing to you in this subject?*

Scenario A

66M with stable angina, type 2 diabetes mellitus (on metformin), asthma (never admitted with it), current smoker (10 pack year history), no alcohol. Fully independent, able to walk around the park unaided outside. Lives with wife in house. No admissions in the last year.

He has been admitted with a urinary tract infection and is currently stable.

He has capacity to make decisions about treatment escalation. It is hospital policy that patients admitted under acute medicine have a documented treatment escalation plan.

Scenario B

79M with ischaemic heart disease (coronary artery bypass graft 3 years ago), atrial fibrillation (on direct oral anticoagulant), type 2 diabetes mellitus (on insulin), asthma (never admitted with it), diverticular disease, ex-smoker (20 pack year history), no alcohol. Has once a day package of care and can mobilise short distances approx. 15m outside with stick, limited by breathlessness; lives in ground floor flat so never has to do stairs. Can get dressed independently but wife waits nearby. One admission in the last year with pneumonia.

He has been admitted with a urinary tract infection and is currently stable.

He has capacity to make decisions about treatment escalation. It is hospital policy that patients admitted under acute medicine have a documented treatment escalation plan.

Scenario C

92M with ischaemic heart disease (coronary artery bypass 16 years ago) and aortic valve replacement, atrial fibrillation (on direct oral anticoagulant), type 2 diabetes mellitus (on insulin), asthma (never admitted with it), diverticular disease, osteoarthritis, hearing impairment (has hearing aids), ex-smoker (20 pack year history), no alcohol. Has a four times a day package of care. He can mobilise to the commode with a Zimmer frame but cannot do stairs. Lives alone. Needs help washing and dressing, can toilet independently. Two admissions in the last year with pneumonia.

He has been admitted with a urinary tract infection and is currently stable.

He has capacity to make decisions about treatment escalation. It is hospital policy that patients admitted under acute medicine have a documented treatment escalation plan.

**Appendix 2**

**Patient study topic guide (2)**

Participants were asked their experience of and imagined response to TEP conversations in an acute hospital setting, ideas about acceptable health outcomes, understanding of treatment options, views on shared decision-making between clinician and patient including balance of decision-making authority, and perceptions of uncertainty around TEP outcomes. A short vignette about an imaginary generally unwell patient going to hospital and being invited to talk about TEP was used to prompt discussion.

**VIGNETTE**

*Jean has been admitted to hospital. She is unwell but expected to recover unless there are complications. The medical doctors say they want to talk to her about what treatments she might receive if she became more unwell and even at risk of dying without extra treatments.*

**INITIAL TOPIC GUIDE**

Thank you for agreeing to participate in this conversation. Today we are going to talk about planning conversations around what should be done if a patient gets more unwell after they have been admitted to hospital. Most people do not become more unwell but some do and then we need to think about different treatments they might have: this includes resuscitation but is also about other treatments like antibiotics and going to intensive care. We will use the scenario you have read as a basis for our conversation.

Everything you say will be confidential and anonymised for the study. Are you happy to proceed?

Initial reactions to the scenario

- *Feelings about the subject being* ***brought up***
- *Familiar or unfamiliar topic; explore any* ***experience*** *of similar conversations*

Ideas about making the treatment escalation planning decision

- *Confidence in* ***knowledge about health*** *and* ***own/loved one’s health conditions***
- ***Knowledge*** *about different treatment* ***options***
  - *Ideas about any specific* ***treatments*** *that are wanted or not wanted*
- *Certainty about what* ***outcomes*** *are acceptable/desirable*
  - *Is the focus on outcomes/goals or specific treatments?*
- *Thoughts on what* ***factors*** *would influence her priorities e.g. media*

Views on being involved in treatment escalation planning

- *Thoughts about patients and clinicians* ***making decisions together*** *about treatment escalation planning*
- *Role of the* ***patient***
  - *Perceived influence and power to participate*
- *Role / expectations of the* ***clinician***
  - *Anticipated* ***contribution*** *of clinician to decision making*
  - *Thoughts about what the clinician is considering e.g* ***external factors***
- *Role of important others e.g.* ***NoK***
- *Expectations of* ***interactions*** *between patient, doctor, others*

Exploring challenging scenarios

- ***Declining*** *recommended treatments*
- ***Demanding*** *non-recommended treatments*
- ***Reconciling*** *differences*

**Appendix 3**

**Data analysis**

First, AK familiarised herself with the data by reading all transcripts and making preliminary notes about cultural and religious influences on decision making. Next, she conducted an inductive, iterative process of semantic and latent coding of self-reported cultural and religious influences on personal decision making. Initial codes were generated from reviewing notes made during data familiarisation. After initially exploring the full dataset for relevant ideas about cultural and religious influences in decision making, a subset of the interviews, where self-reported cultural and religious influences (from any background or belief) were expressed in sufficient depth, informed the final set of codes for analysis. Codes were modified to capture distinct ideas, and new codes were added during coding individual transcripts. AK developed initial themes through mind-mapping. In consultation with the wider research team, themes were then reviewed and further developed by revisiting the original data, and distinct ideas later organised into subthemes for clarity and coherence. In an iterative process which continued during writing up, themes were further refined then defined and named. Interpretations from both clinician and patient datasets were then considered in conjunction in the final analytical stage, during team debriefs and throughout manuscript writing. Coding was completed using the qualitative data analysis software NVivo (QSR International Pty Ltd, release 14.23.2), while theme generation was performed manually.

**Appendix 4**

**Reflexivity**

As an immigrant Egyptian Muslim woman and a UK medical graduate, AK brought a dual perspective to the data. Her medical training provided insight into complexities of TEP decision-making, while her cultural background contributed insight into how cultural and religious factors can influence healthcare interactions. AK was aware that her positionality presented potential biases upon which she reflected throughout the analysis. AK and BEW reflected about how BEW’s background as a White British clinician and interviewer may have influenced interview dialogue with patients and clinicians from various backgrounds about culture and religion.

As a secondary analyst, AK was aware that her lack of direct involvement in the primary data collection might limit appreciation for certain contextual subtleties. AK kept a reflexive journal throughout the analysis and regularly debriefed with the team to ensure all interpretations were grounded in the interview context and aligned with interviewer observations. This approach allowed AK to immerse herself in the data within the primary research context, while bringing an independent analytic perspective.

**Appendix 5**

Extended table of illustrative quotations

| **Theme** | **Quotes** |
| --- | --- |
| **Clinicians theme 1:**  **Decisions informed by a dynamic British medical way** | “So I think why we find this difficult, and why we sometimes find cultural differences difficult, is because we, as doctors in Britain, have also had a very specific cultural upbringing. So what we think is right is what we learnt at medical school, which is, you treat your reversible things, and then when it becomes futile you take out the cannula and you give them morphine. But that is so specific to being in Britain, at this time, being educated in this way. And actually, even compared to our colleagues in Belgium and Holland, who do euthanasia and assisted dying, we’re quite offended by that, by the idea of euthanasia. And we’re also offended by never palliating, so we’re on a very specific spectrum. The same way that the Belgians are quite… More confident about euthanasia and, say, countries that don’t do palliative care are appalled by it. So I think part of the challenge is us feeling that our way, and the way we practice here, and the way we’ve been brought up, and of course our GMC guidance, and our end-of-life guidance, is the only way. And sometimes it can become a challenge to argue that to the patient who’s saying, but it’s not our way, this is not what we want. That’s an ethical challenge. I don’t really know the answer. And sometimes I think, provided the patient isn’t suffering, there can be some leeway into saying, okay, if the family and the patient are absolutely… Sometimes you can say, if they’re that insistent on resuscitation, and there is no harm, and the patient is dead, and it brings peace to the patient to know that they’re getting that, and it brings the family peace to know that they’re getting that, then I don’t actually… Although it makes me feel very uncomfortable as a doctor, in my heart of hearts, it actually doesn’t offend me because it’s just… I know that it’s just against our particular medical model here in the west, that’s very different from the rest of the world.” *– GIM Consultant*  “I've seen it with clinicians who were of the same culture as a family who one would have perceived to be asking for treatments which really are not indicated and won't work. So I think the cultural clash then is between a particular cultural family and more the British medical way. But more and more we're accepting if that's the right word, of the cultural differences and we're trying to support them.” *– ICM Consultant*  “And I think the other element that is quite important and is difficult sometimes to unpick are some of the cultural and religious elements to escalation. And I come from a cultural background where there isn’t a real strong belief in the sanctity of life. And I think most people from the cultural background I’m from would be very interested in quality of life over quantity of life. But that’s not necessarily true for all areas of society. And I think we sometimes struggle with that.”  *– ICM Consultant*  “And so it’s a slightly personal position which I suppose you could argue is a medico-political-ethical position that I’ve taken that our job is to allow a nice death rather than to preserve life at all costs. And I suppose I’m possibly not burdened with a strong religious or cultural obligation to do absolutely everything to preserve life at any cost, maybe, I think.” *– EM Consultant*  “This comes down to, ultimately, personal opinion and your upbringing, your experience as a human, what you think is an acceptable quality of life, and culture. I went to a rehab hospital in Israel, and there were wards of patients in their 80s and 90s on ventilators, and it felt very uncomfortable and wrong. But for that culture it’s not. It’s what they want, and what they would feel is required, or for many people, anyway. So, yes, it’s culture-specific, I suppose, not just individuals.” *– GIM Registrar*  “Perhaps, my understanding is that if this man were in Italy, they probably would take him to ICU. So who knows what the right [thing] to do is.” *– ICM Registrar*  Interviewer: “Where do you think people get a sense of what they would want for themselves?” Participant: “In my experience, it tends to be a lot more from intimate personal experience. So it either tends to be from experience of their own or experience of someone close to them. And often, a lot of the time, that's where it comes from. And then, obviously, you overlay that with sometimes with people's religious beliefs about what's appropriate, what's not appropriate for them.” *– EM Consultant*  Interviewer: “When you’re involving the patient or their family in the discussion, what are you hoping to get from them?” Participant: “So, I think a collateral. So, like an understanding, because I am always very clear. I say look I’m meeting your relative or I’m meeting you in a very different situation from home, so I like to understand who they were two weeks ago or how they were four months ago. So, an understanding of them as a person, their spiritual beliefs or culture they’ve come from and what’s important to them. So, definitely, that kind of thing is important.” *– GIM Registrar*  “But then, I think you still get an idea about the sorts of things that people want to go through or are willing to go through, and then, the things that are important to them. And then, you help guide them about what things will help them achieve what they want to achieve. So, if being independent at home in their own environment is really important, then if they're in multi organ failure, having multiple forms of life support, probably not going to help them achieve that. If watching TV in a nursing home is important to them, and they don't care for food, then actually, if they're insistent that they want to live as long as possible and they're super religious, and they believe they should receive every treatment that man can deliver, then again, relatively straightforward. Although, a difficult situation if you don't think that's right for the patient.” *– GIM Consultant*  “Maybe because I told you it's my first year here in the UK… my point of view maybe a little bit different than what I'm seeing here…. He is 70, he's an old patient, has a lot of multiple comorbidities, and very low functional capacity, even if his daily activities, can dress independently, but wife waits nearby. But still, for me, he's a human being, fully conscious, maybe he wants to have another two, three years of living. So with this patient I will discuss and I will tell him that, if things got complicated, I will tell him that the treatment may be more painful, and may be more useless, but I will give him that choice.”*– ICM Registrar*  “And as a consultant, I certainly think I'm more soft, I'm certainly softer than a lot of my colleagues and I would admit patients that my colleagues wouldn’t admit. And I think the fact religious beliefs, for me, is a big factor, and so I often feel like we don’t have the decision to make God’s decision, and so I'm very religious and that is a big factor for me.” *– ICM Consultant*  “But we’re constantly living in fear that our colleagues either think we’re murderers, or nihilists, or over-investigators. And so we feel the need to fit in with the model around us, especially… And of course, we have our guidance, we have our GMC and our good practice, so we know the structures in which we need to work. But I think, sometimes, deviating from that can feel quite naked.” *– GIM Consultant* |
|  | |
| **Clinicians theme 2:**  **Cultural and clinical dissonance creates tension in TEP discussions** | “The big differences happen I think when people have their religious or cultural beliefs. I'm trying to phrase it correctly, but. So strong that they can't see the wood for the trees. And so they just get a line almost from their cultural backgrounds that one must do everything no matter what, without understanding the clinical picture as well. And there are times when I'm surprised when I start to explain to families about someone's situation and they go, well, of course, you're not going to do resuscitation and chest compressions and put them on a ventilator. She's dying. So yes. But on the whole, I think if I do a good enough job of explaining why or why not I propose a particular treatment plan then families are accepting of that.” *– ICM Consultant*  “And there comes then an interesting discussion with them about how much we can actually physically do to give them that, grant that wish, that want, and how much is simply beyond our capability. And that often comes up with a religious discussion with some people who are very, very embedded, either major religion or innate religious beliefs, that they have to take every single opportunity to prolong life regardless of what that life is, so our concepts of quality of life or autonomy don’t really come into it.” *– PC Consultant*  “But I think it’s a lot of cultural, religious, and wider determinants. For example, in [a hospital], where I was working previously, mostly, people, they’re not white British. They’re usually South-East Asian or from the Muslim countries, and they just have very different perceptions of what doctors should be doing, and what the medicine should be doing. They don’t think about it as not doing anything as a good thing. That’s the opposite of what they think. So it’s very hard to change a cultural belief.” *– EM Registrar*  “For example, when I'm speaking with Arabic family it's hard to deliver bad news. I'm Arabic but I'm sorry to say that they don't believe that there is a limitation for medicine. They think that people are like machines and whatever we do we should have a good effect. So delivering bad news, or that we will palliate or we will stop giving the medical treatment is not that easy. That's why when I was working before in Arabic country we will just say giving more medication or intubation or CPR will cause more harm, but the decision will be them. And we will not insist in pushing them toward any decision. Because they are not… I don't know how to say it in English, but they believe that everything we do should have an effect, like, yes.” *– ICM Registrar*  Interviewer: “I was interested, a while back you were saying about, if you had your ten patients on the take, there’ll be X number where it’ll be really difficult, and there’ll be a considerable proportion where, actually, they’re really fine with having that escalation, the conversation. And I wonder, what do you think it is about those patients that makes them okay with having it?” Participant: “I think there probably is a cultural element to it, and a religious element, and an… So an element of culture, an element of religion, and an element of the extent of family involvement, and how much the patient wants the family to be involved. And I think, sometimes, there are situations where the patients, and even a medical relative, might say, we think that palliation might be more appropriate, but culturally… And the wider family don’t. They are actually easier conversations, because at least you can have the more nuanced discussion. As opposed to the people that say, do not play God, do not kill my mum, and they’re often more challenging.” *– GIM Consultant*  “No, I think just… Yes, and culturally I think there are just some cultures where, regardless of the religion, say… People, I’ve noticed, from the Middle East, whether they’re Christian, Jewish or Muslim, will have certain opinions in terms of the family have the… As you were saying before, have the strong decision-making ability, and the patients will often defer to the family. And that the idea of death is still… And sometimes people will say, actually, I know but I need to advocate for my mum, and therefore I need to keep arguing with you for full resus because that, for me, is advocating for my mum.” *– GIM Consultant*  Interviewer: So I think many of us are just about all right with saying, this patient has a view which is different from mine, but I will respect it because I must. But when the patient’s view is that they will defer to their family, and then the family say they want something that is different from what we would want, I wonder if that, does that then take it a step too far? Because still, in theory, that is what is most important to the patient, but I think I feel we struggle from that.” Participant: “I agree, and I think that’s so far from our medical upbringing, and from what we believe is the right thing. Where, of course, we have to follow the GMC, which is the patient is the most important person to you. I agree, I find it very jarring.” *– GIM Consultant*  “I think they’re difficult. But I think that’s where trying to identify where, which I don’t think that we often state very clearly, saying to someone, actually, I think what this is, is you’re dying. And then that is… There’s nothing we can do to reverse it. It’s very different to saying you’ve got a disease where there’s a good chance that you won’t survive even if we do this. The problem is if there is a chance that you’d survive, even if it’s a much-reduced quality of life, and that’s what the patient would want, I find it difficult to not offer that. Because why wouldn’t we offer that? What is it that we’re losing out on by not offering that? If they feel that they would want it, it’s resources. But I’m not sure we’re the people who should be limiting resources. Because if resources should be limited and inform those decisions, there ought to be clear guidance how we do that. So if you think there is some chance of recovery, and the family say, we would be… Or the patient, more importantly, says, yes, I would be happy to be alive at any cost. Then I think it’s difficult not to offer it.” *– ICM Consultant, in the context of patients asking for treatment escalation on religious grounds*  “If you bring someone who they're going to ventilate for the next three months and then die, that doesn’t keep morale very good. And so that also, along with cost, is important to take into account when you're making these kind of decisions. But I think, for me, on a personal level, with my religious belief, I make sure that doesn’t interfere in my decision making. I know those are important factors, but I don’t let them… However, there are important things to also consider.” *– ICM Consultant*  “Well, I think you see that in the States already, don’t you, where people can pay for it and it doesn’t mean their outcomes are any better, which is reassuring, isn’t it actually? Because often I can say, in other countries, people would have done this, but actually we know that their outcomes are no better. I don’t make it about resource when I talk to families, but I think it should be in the back of our heads because actually I sometimes look at our ITU and think, oh my God, there’s a lot of people that I’m surprised are in the ITU.” *– PC Consultant* |
|  | |
| **Clinicians theme 3:**  **Convincing and compromising: negotiating a culturally acceptable decision** | “I would try and challenge some of it, and talk to him about life being sacred and try and find out if he’s... It sounds like he's religious. Try and offer some religious support. Try and ask him if he was worried about dying. I think you try and explain to the patient that an attempt to chest compressions is not what resuscitation is. Either you do it properly and you fully resuscitate someone or you don't, and that it's a medical decision to treatment, like any other treatment. But we want him to understand the rationale on which the treatment is based or the decision is based.” *– GIM Consultant*  “I really don’t think there’s a clear answer to that. I think it’s about having that discussion as much as possible, revisiting it again at a later date, giving people some time to think over it, and discuss it with their family, offering them an opportunity for a second opinion. I don’t know whether we have this. If it’s religious views that are holding them back, then offering them the chance to speak to chaplains and so on about this. But it’s a tricky scenario. And I think the outcome may still be very similar in the sense that you do give it a halfway house approach. And if they end up in that scenario, you’d try CPR for a bit and then call it off after a couple of cycles.” […] “I think we generally do have a gut feeling one way or the other about these things. But ultimately I think we leave it, not leave it up to the patient, but achieve a compromise as best as possible, if there’s a strong conflict. But I don’t think we necessarily change our minds about it.” *– GIM Registrar*  “I think it’s also to get a feel what the family think. Because say that I were to still exercise my decision, I’d want to know what the family would feel at the end of it all. If the family also hold really strong opinions like him, then I think it goes back to the point I was saying earlier about not wanting to break down that whole doctor patient relationship for the long run. So, then I think we may have to end up achieving a halfway house. But I think if we’re able to explain to the family, and they understand, then they may be able to explain to the patient what exactly we’re trying to say. And then it comes from a place where it’s someone who cares for them telling them that. So, I think the aim is still to convince the patient that this is not a good idea by involving more people saying the same thing. *– GIM Registrar, in response to a question about involving family in decision-making for a capacitous patient requesting further escalation on religious grounds*  “I push back a little and I would say, can we speak to a religious person like an imam or, I don’t know, a chaplain or that sort of thing. I think if it were just me and him, then I think that would be a conversation that we can have, leave it between us, there’s no need to escalate. But then if the family are going to make the hospital and the junior doctors and the staff nurse’s lives very difficult and there’s a chance that it’s going to get out of hand, unfortunately it isn’t just the relationship between the patient, it becomes the whole hospital, NHS reputation, then you’d have to escalate to someone, the medical director or whatever.” …. “But I would say no. If I was the medical director, I would say, I’m sorry, this is not something we would do.” *– GIM Consultant*  Interviewer: Would you ever say, we’ll leave you for full escalation, we’ll leave you for everything, because this is what you want, or do you think it just really doesn’t sit well?” Participant: “It doesn’t sit with me, no. I would get other people on board to support my decision, but I wouldn’t do it.” Interviewer: “What makes you feel that way?” Participant: “Because I would be harming the patient and I would be doing something that doesn’t sit well with me, doesn’t sit comfortably.” *– GIM Consultant, in the context of conflict with a patient requesting further escalation on religious grounds*  “So, I think we for want of a better word, the more professional use of the word, we respect [cultural differences] and therefore we try and support families when we feel that somebody is in a palliative care situation. So, if somebody's come in intubated yet they've had a catastrophic brain injury and they're a frail 88-year old but for some reason, they've been intubated and if the family can't accept that then we give them time. But you just say to them, I'm not going to increase the organ support. There's a chance that the organs could stop. There has to be a line where you go, I'm not going to do this, I'm not going to do that. But if something has already been started then you give time and support. I often say we'll probably forget this particular patient and their family because we do this so much but they'll always remember and so, therefore, you end up changing the way that you are. You're supporting the patient to a degree but actually, you're supporting the family in the end-of-life process. […] But yes, I think the way that we have moved from, I don't know, decades ago is that we will offer some support in order to give time for families to accept. And that's usually what people need is time.” *– ICM Consultant*  “I think that’s really hard. I definitely culturally had family and patients say in our religion, life is life at all costs. So, we want to be kept alive and we want all the treatment possible. I think that is a very difficult situation. I definitely know many situations where that’s happened. The patient does remain for full escalation in the recognition that it’s unlikely to be successful, but the conflict between patient, relatives, medical team is so significant that actually by pushing that you’re then breaking down any relationship you have with that individual.” *– GIM Registrar* |
|  | |
| **Patients theme 1:**  **A relational decision made within a family network** |  |
| Subtheme 1: Making a personal decision is an interpersonal process | “But sometimes they don't. Their brain, this capability of thinking is gone because of the medications and because of their illnesses and everything. I think this is when, I think, in our custom, in our Indian culture, generally they are the sons. They try to talk to the sisters and everyone, and they try and make a decision.” - *Female, aged 75-84, mild frailty, Indian ethnicity*  “They can talk to the patient, depending on how acceptable the patient is, how strong and willing the patient is. If you notice that the patient is not very strong to accept the proposals, then it’s better to talk to the family. In most cases, I’ve seen that patients who are deteriorating, doctors talk to the family. Yes, this is what we suspect, this is what is going to be happening, so this is what we are prepared to do. What do you say? Then the family, the key person in the family, they discuss it with the patient. This is somebody the patient has confidence in, that is close to the patient, would talk to her or him or would agree that the doctors communicate directly with the patient. They would say, oh, no, you talk to her, she would rather understand when you talk than me…” *– Female, aged 65-74, no frailty, African ethnicity*  “It’s a hard conversation, and it’s a conversation that we do better through a family member, through a very strong family member.” *– Female, aged 65-74, no frailty, African ethnicity*  “Personally, if there are more than one adult in the family, they should sit down and they should make a decision. Because they don't want to blame the doctors for the rest of their life that he killed my mother or he killed my father. So this is my opinion. Doctors, as I said earlier to you, they always explain what is happening. And they always say pros and cons. This can happen. That's how they speak. This can happen. I'm not saying it will happen. So I think the final decision has to be... the family.” *- Female, aged 75-84, mild frailty, Indian ethnicity* |
| Subtheme 2: Familial caregiving as a cultural ideal | “At least I have got five or six telephones calls every week from my family, encourage me to live on. Come on, we are going to see you, we are missing you, this and that. That motivates me to live, and use my medicine properly, and get well and go home for a few days or a few weeks. That’s very important. But if you are alone in this country, you have got your wife and two kids, that’s it, you don’t have the whole family around you… And we are very family-orientated compared with here” *– Male, aged 75-84, moderate frailty, Any other ethnic group*  “You have come to the Intensive Care the whole week, it’s going to two weeks, your condition remains the same. The family is always important in such cases. Even the patient is always important in such cases, as long as the patient can take a decision whether to continue to stay in the Intensive Care or to go out to the ward. Because some people feel better when there are people around them. So when you restrict that, they don’t improve. They themselves wonder, oh, what is wrong with me? But I’m not improving. I’m getting all the medication, but I’m not improving. Maybe I’m missing something. Family. People coming to visit.” *– Female, aged 65-74, no frailty, African ethnicity*  Husband: “Family encouragement is very important, that motivates you to live.” Wife: “Yes, but not carers.” *– Husband: Male, aged 75-84, moderate frailty, Any other ethnic group. Wife: Female, aged 75-84, mild frailty, Any other ethnic group*  “It depends how much family support he has at the back. Nowadays, nobody cares. If somebody asks, they might give him one or two and then they get fed up if you are in the bed. Say he’s living with his son and daughter or his wife, whatever, they might respond to his request if there are a few. They cannot do it for all of their life. They will get fed up and then they try to dump him in the carer’s home.” - *Male, aged 65-74 years, moderate frailty, Indian ethnicity*  **Participant:** “Saying that, I know that you do get sometimes nurses who go and put the medications and everything. But, I don't think that when the nurses go, they are that welcome in any families. It's always... First few days are fine. But when the nurse or whoever is coming for helping every day, it seems that, oh, maybe she knows too much about the family...” **Inteviewer**: “It's an invasion of privacy.” **Participant**:” Exactly. And that happens in our Indian family a lot. And this is not a prejudice statement …. This is just a statement. Not just our Indian people, any Eastern people from Pakistan. Because that's our culture.” *- Female, aged 75-84, mild frailty, Indian ethnicity*    “Yes. And you know and I know, when the carer comes, and I'm telling you from my own experience, they are lovely people, but they can only go so far. They can only go by the books. And otherwise, they will not. So we actually did more for our parents than the carer came, but my father will not allow the carer to touch him. So it is such a vast subject, how to give the patient the best.” *- Female, aged 75-84, mild frailty, Indian ethnicity* |
|  | |
| **Patients theme 2:**  **Expectations built on experiences in different contexts** | “Yes, I can do both. I still trust the doctor. I trust what they’re doing and that is why, in the Western World, we have this opportunity of the expert helping you, prolonging our lives […]. And a number of people I know, they’ve died, third world country or these things, all because of the health we get here.” - *Male, aged 75-84, no frailty, African ethnicity*  “Even in the Philippines general hospital, I worked in the charity ward. You can’t imagine people sometimes. They don’t have money to buy. And you know the supply, we are the government hospital. We are not like NHS. You have everything. But still we give chance.” “But you have to do your best to let them survive. And sometimes they survive. Not only sometimes, most of them survive.” *– Male, aged 65-74, no frailty, Any other Asian background [previous nurse in home country]*    “It's very difficult to decide. And I'm so happy that in this country, doctors don't give up until the patient gives up. They try all the things that are there to save the person. and you can feel very proud of them. Honestly, they really go out of their way to make him better and do things for them. But sometimes you feel that, no, let the patient go because the patient will be a cabbage.” *- Female, aged 75-84, mild frailty, Indian ethnicity* |
|  | |
| **Patients theme 3:**  **Expert decision, Divine outcome** | “Well, I think it’s right because they’re specialist and expert in their own field. If I get to that stage and they give me, I would take it. And as I’ve said, as a Christian, I have alternative solution, an alternative, I would take it because nobody wants to die really. I go to that treatment. If it improve my health, I happy. If it doesn’t, turn to my spiritual belief. I will use it. I won’t turn anybody down when you get to that stage.” - *Male, aged 75-84, no frailty, African ethnicity*  **Interviewer**: “Do you mean that lots of Jewish people might think it's an important conversation, or did I misunderstand?” **Participant**: “No, I can't think that some people would ignore advice from doctors. We treat them as reliable, sensible people.” *– Female, aged 85+, moderate frailty, Any other White background*  “As far as my religion is concerned, they know we have to go one day. It doesn’t matter if you live 60 years or 100 years. One day is decided. It doesn’t matter even if you are in hospital. It doesn’t matter if you see a good consultant or a very famous one, when the time comes it’s up. Time to go. Nobody will save you.” - *Male, aged 65-74 years, moderate frailty, Indian ethnicity*  “[Faith plays] a very important role. In fact, I believe in God more than I believe in the medicine. He cured me not the medicine, the medicine’s the middle-man. The doctors are the middle-men, they deliver the medicine to you in order to cure you. But he gives you the green light, not the doctor, not the medicine. But if you give… If you believe in God you will be cured, it doesn’t matter what sort of illness you have. Even if you have got cancer you will be cured if you believe in him. And I do believe in him, that’s why I used the medicine as a middle-man, as a middle-item.” *– Male, aged 75-84, moderate frailty, Any other ethnic group*  “If He wants to take me, He will take me. If he wants to cure me, He will cure me. He will cure me through you, through the doctor.” – *Female, aged 75-84, mild frailty, Arab ethnicity*  “The Almighty doesn’t ask are you happy with my decision or do I want you back.” *– Female, aged 85+, moderate frailty, Indian ethnicity*  “It was His [God’s] decision to take me, not the doctor.” *– Male, aged 75-84, moderate frailty, Any other ethnic group, in the context a ‘second opinion’ clinician agreeing on limiting treatment escalation*    “We believe, this is our faith. We believe that you, as a doctor, has got to help as much as possible. But not when there is no solution, and keeping this machine. I don’t believe in this.” *- Female, aged 75-84, mild frailty, Arab ethnicity*  **Interviewer**: “What would be your perspective from a faith point of view, on that? Is that worth doing, or not?” **Participant**: “No […] If you are certain, as a doctor, that if you try this machine, and I will still be a vegetable, or not conscious at all, no, you shouldn’t do it, no.” *- Female, aged 75-84, mild frailty, Arab ethnicity*  “He should have the right to live his life. The doctors should not be the God power to decide this person should die and this person should live.” *- Male, aged 65-74 years, moderate frailty, Indian ethnicity*  “Let them decide. Because keeping the person, I think, is just… Although we pray and pray, and that’s the last resort, the prayer. That’s what most of us believe in, that we will pray, and then we’ll come back to life. But if you’re coming back to life, and your life is not worth it, so I don’t think you should come back to life just to give your family more stress, more agony, in seeing you in that condition.” *– Female, aged 65-74, no frailty, African ethnicity*  “You can be young. It can be old age. You have to leave this place. But what I always pray for myself is that Allah, I mean, God should take me without being dependent on others for my day-to-day living.” *– Male, aged 65-74, moderate frailty, Indian ethnicity*  “It’s not a religious thing, but it is something to do with your faith. We say, leave it to Allah, leave it to God, He will take care of me. That’s what we believe. So this is how I will think of it.” - *Female, aged 75-84, mild frailty, Arab ethnicity*  “If you have spiritual belief and you know that there is someone who can pray for you and it does work. And that problem you have will disappear. So I believe in God Almighty. I believe because as I’ve said, I read Bible every day. I read it.” - *Male, aged 75-84, no frailty, African ethnicity*  “We live for our beliefs, and faith, as much as we live for our children, and family, and friends. You have to have a belief, if you don’t have a belief you have nothing.” – *Female, aged 75-84, mild frailty, Any other ethnic group*  **Interviewer**: “Is that something every Muslim thinks, or actually…?” **Participant** “No, no. This depends on the person. We believe that when the time comes for you to die, it’s finished. We have a saying in Arabic, it says reasons are a lot, but the death is there.” - *Female, aged 75-84, mild frailty, Arab ethnicity* |

**References**

1. Warner BE, Wells M, Vindrola-Padros C, et al.; Shared decision-making with older people on TReatment Escalation planning for Acute deterioration in the emergency Medical Setting: a qualitative study of Clinicians’ perspectives (STREAMS-C). *Age and Ageing* 2024;**53**(9). doi: 10.1093/ageing/afae204.

2. Warner BE, Wells M, Vindrola C, et al.; Shared decision making with older people on treatment escalation planning for acute deterioration in the emergency medical setting: a UK-based qualitative study of patient perspectives (STREAMS-P). *The Lancet Healthy Longevity* 2025;**6**(3). doi: 10.1016/j.lanhl.2025.100689.
